# Impact of COVID-19 on viral respiratory infection epidemiology in young children: A single-center analysis

**DOI:** 10.3389/fpubh.2022.931242

**Published:** 2022-09-20

**Authors:** David Paul Shen, François Vermeulen, Anne Debeer, Katrien Lagrou, Anne Smits

**Affiliations:** ^1^Faculty of Medicine, KU Leuven, Leuven, Belgium; ^2^Department of Pediatrics, University Hospitals Leuven, Leuven, Belgium; ^3^Department of Development and Regeneration, KU Leuven, Leuven, Belgium; ^4^Neonatal Intensive Care Unit, University Hospitals Leuven, Leuven, Belgium; ^5^Department of Laboratory Medicine and National Reference Centre for Respiratory Pathogens, University Hospitals Leuven, Leuven, Belgium; ^6^Department of Microbiology, Immunology and Transplantation, KU Leuven, Leuven, Belgium; ^7^L-C&Y, KU Leuven Child & Youth Institute, Leuven, Belgium

**Keywords:** COVID-19, respiratory infection, epidemiology, children, influenza, RSV, non-pharmaceutical interventions

## Abstract

**Background:**

The COVID-19 pandemic impacts different health aspects. Concomitant with the adoption of non-pharmaceutical interventions (NPIs) to reduce the spread of SARS-CoV-2, global surveillance studies reported a reduction in occurrence of respiratory pathogens like influenza A and B virus (IAV & IBV) and respiratory syncytial virus (RSV). We hypothesized to observe this collateral benefit on viral respiratory infection epidemiology in young children.

**Methods:**

Respiratory samples of children aged below 6 years, presenting at the outpatient clinic, emergency department, or pediatric infectious diseases department of the University Hospitals Leuven, between April 2017 and April 2021 were retrospectively analyzed. The occurrence (positivity rate), and seasonal patterns of viral respiratory infections were described. Chi-squared or Fisher's exact test (and Bonferroni correction) were used to explore differences in occurrence between 2020-2021 and previous 12-month (April to April) periods.

**Results:**

We included 3020 samples (453 respiratory panels, 2567 single SARS-CoV-2 PCR tests). IAV and IBV were not detected from March and January 2020, respectively. For IAV, positivity rate in 2020–2021 (0%, *n* = 0) was significantly different from 2018-2019 (12.4%, *n* = 17) (*p* < 0.001) and 2019-2020 (15.4%, *n* = 19) (*p* < 0.001). IBV positivity rate in 2020-2021 (0%, *n* = 0) was not significantly different from previous periods. RSV occurrence was significantly lower in 2020–2021 (3.2%, *n* = 3), compared to 2017-2018 (15.0%, *n* = 15) (*p* = 0.006), 2018–2019 (16.1%, *n* = 22) (*p* = 0.002) and 2019-2020 (22.8%, *n* = 28) (*p* < 0.001). The RSV (winter) peak was absent and presented later (March-April 2021). Positivity rate of parainfluenza virus 3 (PIV-3) was significantly higher in 2020-2021 (11.8%, *n* = 11) than 2017-2018 (1%, *n* = 1) (*p* = 0.002). PIV-3 was absent from April 2020 to January 2021, whereas no clear seasonal pattern was distinguished the other years. For the other viruses tested, no significant differences in occurrence were observed between 2020-2021 and previous periods. From March 2020 onwards, 20 cases (0.7%) of SARS-CoV-2 were identified.

**Conclusion:**

These findings reinforce the hypothesis of NPIs impacting the epidemiology of influenza viruses and RSV in young children. Compared to previous periods, no IAV and IBV cases were observed in the 2020-2021 study period, and the RSV peak occurred later. Since the pandemic is still ongoing, continuation of epidemiological surveillance, even on a larger scale, is indicated.

## Introduction

The COVID-19 pandemic has a large impact on different health aspects. Preventative measures such as lockdowns, social distancing, the use of face masks, and increased awareness on hand hygiene have been adopted widely to reduce the spread of the formerly unknown SARS-CoV-2 (1). These non-pharmaceutical interventions (NPIs), also known as public health measures or community mitigation strategies, are believed to be effective against the transmission of other respiratory infections as well (2).

Flu-like symptoms trigger a significant proportion of primary care visits (3). Furthermore, acute respiratory infections are major causes of morbidity and mortality in the youngest and eldest populations (4–9). In the absence of a pandemic, seasonal peaks of influenza A and B virus (IAV & IBV), respiratory syncytial virus (RSV), and common coronaviruses typically occur during the winter season (10, 11). Parainfluenza virus (PIV), human metapneumovirus (hMPV), adenovirus (ADV), rhinovirus (RV), and bocavirus circulate throughout the year, with usually higher infection rates during winter (10, 11). Compared to other age categories, children below six years have a higher incidence and vulnerability for viral respiratory infections (11). Especially RSV carries a significant hospitalization rate (12, 13). In contrast, with COVID-19, children are more often asymptomatic or have a milder disease course (14–16).

Concomitant with the implementation of NPIs to prevent the spread of SARS-CoV-2, the WHO European Region Influenza Surveillance network reported an abrupt end of influenza activity in March 2020, earlier than previous seasons (17). Likewise, the influenza incidence in the United States decreased abruptly within two weeks of the national COVID-19 emergency declaration in March 2020 (18). Also afterwards, global surveillance studies confirmed significantly decreased influenza activity (2, 18–24). Several retrospective studies reported similar findings for other respiratory pathogens, including but not limited to RSV, PIV, and hMPV (24–35).

We hypothesized that the public health measures adopted to reduce the spread of SARS-CoV-2 have a collateral benefit on the occurrence of other respiratory infections. Therefore, the objective of this study was to determine the impact of COVID-19 on the epidemiology of respiratory infections in young children in a single, Belgian university hospital.

## Materials and methods

### Study population

This retrospective analysis describes the occurrence and seasonal patterns of respiratory infections in young children over a 4-year period (April 16th, 2017, until April 15th, 2021). Inclusion criteria were as follows: children below 6 years of age who presented at the outpatient clinic, the emergency department, or the pediatric infectious diseases department of the University Hospitals Leuven, Belgium, within the predefined period and had at least one respiratory sample collected. Indication for sampling can be respiratory infection (respiratory panel) or SARS-CoV-2 screening (isolated SARS-CoV-2 PCR). The epidemiology and etiology of respiratory infections in this well-defined age category have been described in detail by the scientific community and were therefore selected (11, 13, 31, 36). Ethical approval has been granted by the Educational-Support Committee of the Research Ethics Committee UZ/KU Leuven and the data was pseudonymized prior to analysis.

### Data collection

The retrospective data search was based on a query performed by the Department of Laboratory Medicine of the University Hospitals Leuven, Belgium, in the electronic laboratory system. The data collected for analysis include age (years), sex (male/female), results of respiratory sampling, types of respiratory sampling (oro- or nasopharyngeal swab, sputum, bronchoalveolar lavage, bronchial or endotracheal aspirate), and date of sampling. Viral (*n* = 24), bacterial (*n* = 6), and fungal (*n* = 1) pathogens were identified using the standard respiratory pathogen panel of the University Hospitals Leuven (37, 38). The test method consists of DNA/RNA-extraction using NucliSens® easyMAG®, followed by real-time polymerase chain reaction (PCR) TaqMan® (37). Because of its poor specificity to differentiate between rhinoviruses (RV) and enteroviruses (EV), the test method was modified to a combined PCR for human rhinovirus/enterovirus (HRV/ENT) in January 2018. For comparability, data of RV and EV before January 2018 were merged. From January 2020 onwards, SARS-CoV-2 was included in the respiratory pathogen panel, and was in addition available as an isolated PCR test. The dataset was provided as a Microsoft^®^ Excel® file. As the focus of current study was analysis of (non-SARS-CoV-2) viral pathogens, data of respiratory panels were selected. For analysis of SARS-CoV-2 occurrence, both results of respiratory panels and isolated SARS-CoV-2 tests were used.

### Statistical methods

Descriptive statistics were performed. The 4-year study period was divided into four periods of 12 months (e.g., 2017-2018), starting on April 16th and ending on April 15th of the following year. Compared to calendar years (January-December), this allows that the periods each contained a full winter season. The absolute number of positive tests per month or year per identified pathogen was defined as monthly and yearly occurrence respectively. The positivity rate per pathogen (in percent) was calculated by dividing the number of positive tests by the total number of tests performed. For graphical representation, the positivity rate was expressed as a decimal number. The Chi-squared or Fisher's exact test was used to explore differences in pathogen distribution across the four periods. A *p*-value < 0.05 was considered statistically significant. The Fisher's exact test was used when at least one of the values in the 2 x 2 contingency table was smaller than five. To correct for multiple testing after comparison of the period 2020-2021 to the previous periods (i.e., 2017-2018, 2018-2019 and 2019-2020), the Bonferroni correction was applied with an adjusted *p*-value < 0.01 considered significant. To achieve a general overview on distribution of respiratory pathogens during the study period for the different ages, the number of positive tests of a specific pathogen was divided by the total number of positive tests for each age group and provided as percentage (%). As pathogen distribution by age was not the main objective of the current study, and due to the anticipated limited number of positive tests in some of these subanalyses, no further between-age group and between-period explorations were performed. Statistical analysis was performed using MedCalc® Statistical Software, version 20.015 (MedCalc Software Ltd, Ostend, Belgium), and IBM® SPSS® Statistics for Windows, version 28.0.1.0 (IBM Corp., Armonk, New York, USA).

## Results

### Study population characteristics

**Figure 1:**
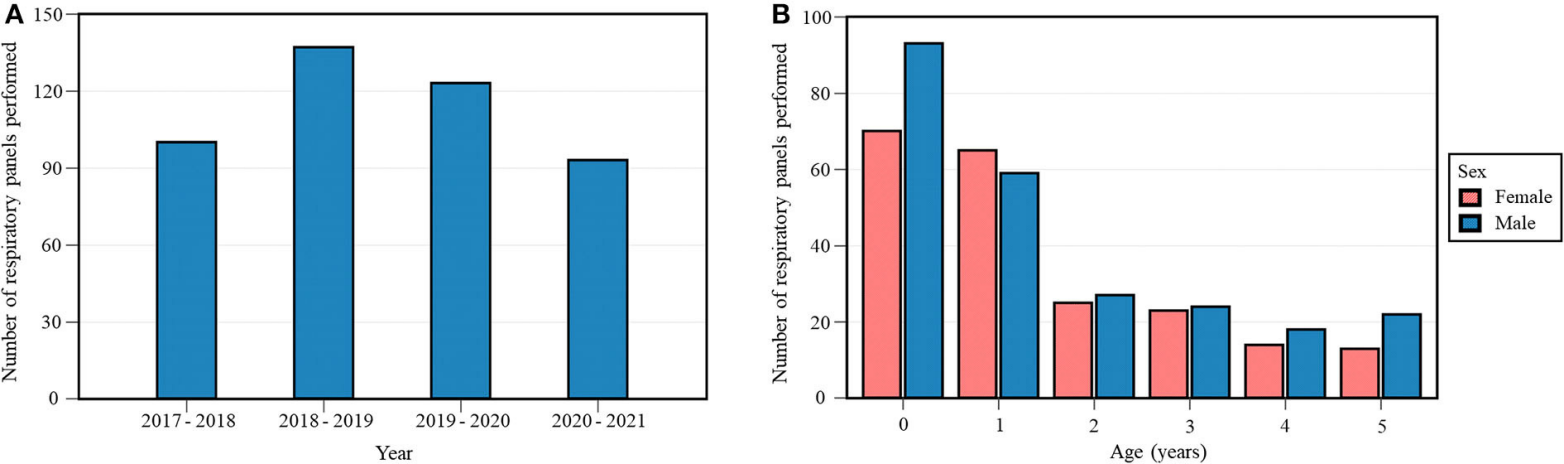
Annual number **(A)** and study population characteristics (age, sex) **(B)** of respiratory panels performed during the study period. Each 12-month period started on April 16th and ended on April 15th of the following year.

Out of 3020 respiratory samples (453 respiratory panels and 2567 SARS-CoV-2 PCR tests) in the dataset, the 453 respiratory panels of 411 patients aged 0–5 years were selected for further investigation (Table 1, Figure 1A). Hence, some patients had multiple respiratory samples collected over the years, which were all considered as individual infection episodes. Detection of SARS-CoV-2 was included in the 131 respiratory panels performed since January 2020. Respiratory sampling over four years included 3018 oro- and nasopharyngeal swabs and two bronchoalveolar lavages. Study population characteristics of the respiratory panels are presented in Table 2 and Figure 1B.

**Table 1:** Total number of respiratory samples included in this study.

	**2017–2018**	**2018–2019**	**2019–2020**	**2020–2021**	**Total**
Number of respiratory panels	100	137	123	93	453
With SARS-CoV-2 included	0	0	38	93	131
Number of SARS-CoV-2 PCR tests	0	0	96	2471	2567
Total number of respiratory samples	100	137	219	2564	3020

The distinction was made based on the analysis performed: a respiratory panel or an (isolated) SARS-CoV-2 PCR test. Detection of SARS-CoV-2 was included in the respiratory panel starting January 2020.

**Table 2:** Study population characteristics of respiratory panels performed: age and sex.

	**Age (years)**						
**Sex**	**0**	**1**	**2**	**3**	**4**	**5**	**Total**
**Female**	70	65	25	23	14	13	210 (46.4%)
**Male**	93	59	27	24	18	22	243 (53.6%)
**Total**	163 (36.0%)	124 (27.4%)	52 (11.5%)	47 (10.4%)	32 (7.1%)	35 (7.7%)	453

The emergency department was the hospital unit with the highest number of respiratory panels performed per year (Supplementary Figure S1A). The age distribution of respiratory panels performed per hospital unit is represented graphically in Supplementary Figure S1B.

Species names of the included pathogens are provided in Table 3.

**Table 3:** Annual number and percentage of positive tests for each investigated respiratory pathogen.

		**2017–2018**	**2018–2019**	**2019–2020**	**2020–2021**	**Total**
Influenza A virus	Positive tests / total tests	4 / 100	17 / 137	19 / 123	0 / 93	40 / 453
	Positivity rate	4.0%	12.4%	15.4%	0.0%	8.8%
	Significance level	*p =* 0.122	***p** **<*** 0.001*	***p** **<*** 0.001*		***p** **<*** 0.001*
Influenza B virus	Positive tests / total tests	4 / 100	0 / 137	2 / 123	0 / 93	6 / 453
	Positivity rate	4.0%	0.0%	1.6%	0.0%	1.3%
	Significance level	*p =* 0.122	/	*p =* 0.507		***p** **=*** 0.020*
Respiratory syncytial virus	Positive tests / total tests	15 / 100	22 / 137	28 / 123	3 / 93	68 / 453
	Positivity rate	15.0%	16.1%	22.8%	3.2%	15.0%
	Significance level	***p** **=*** 0.006*	***p** **=*** 0.002*	***p** **<*** 0.001*		***p** **<*** 0.001*
Parainfluenza virus 1	Positive tests / total tests	2 / 100	0 / 137	5 / 123	0 / 93	7 / 453
	Positivity rate	2.0%	0.0%	4.1%	0.0%	1.5%
	Significance level	*p =* 0.498	/	*p =* 0.072		***p** **=*** 0.021*
Parainfluenza virus 2	Positive tests / total tests	1 / 100	1 / 137	1 / 123	0 / 93	3 / 453
	Positivity rate	1.0%	0.7%	0.8%	0.0%	0.7%
	Significance level	*p =* 1.000	*p =* 1.000	*p =* 1.000		*p =* 1.000
Parainfluenza virus 3	Positive tests / total tests	1 / 100	13 / 137	5 / 123	11 / 93	30 / 453
	Positivity rate	1.0%	9.5%	4.1%	11.8%	6.6%
	Significance level	***p** **=*** 0.002*	*p =* 0.570	*p =* 0.031		***p** **=*** 0.004*
Parainfluenza virus 4	Positive tests / total tests	1 / 100	3 / 137	2 / 123	0 / 93	6 / 453
	Positivity rate	1.0%	2.2%	1.6%	0.0%	1.3%
	Significance level	*p =* 1.000	*p =* 0.274	*p =* 0.507		*p =* 0.643
Adenovirus	Positive tests / total tests	26 / 100	29 / 137	16 / 123	15 / 93	86 / 453
	Positivity rate	26.0%	21.2%	13.0%	16.1%	19.0%
	Significance level	*p =* 0.095	*p =* 0.341	*p =* 0.518		*p =* 0.073
Bocavirus	Positive tests / total tests	10 / 100	13 / 137	6 / 123	9 / 93	38 / 453
	Positivity rate	10.0%	9.5%	4.9%	9.7%	8.4%
	Significance level	*p =* 0.940	*p =* 0.962	*p =* 0.171		*p =* 0.444
Human metapneumovirus	Positive tests / total tests	5 / 100	9 / 137	3 / 123	4 / 93	21 / 453
	Positivity rate	5.0%	6.6%	2.4%	4.3%	4.6%
	Significance level	*p =* 1.000	*p =* 0.569	*p =* 0.467		*p =* 0.461
Human rhinovirus/enterovirus ^†^	Positive tests / total tests	45 / 100	56 / 137	59 / 123	44 / 93	213 / 519
	Positivity rate	45.0%	40.9%	48.0%	47.3%	41.0%
	Significance level	*p =* 0.748	*p =* 0.335	*p =* 0.924		*p =* 0.663
Rhinovirus	Positive tests / total tests	27 / 66	/	/	/	27 / 66
	Positivity rate	40.9%	/	/	/	40.9%
	Significance level	/	/	/		/
Enterovirus	Positive tests / total tests	13 / 66	/	/	/	13 / 66
	Positivity rate	19.7%	/	/	/	19.7%
	Significance level	/	/	/		/
Coronavirus 229E	Positive tests / total tests	0 / 100	2 / 137	0 / 123	0 / 93	2 / 453
	Positivity rate	0.0%	1.5%	0.0%	0.0%	0.4%
	Significance level	/	*p =* 0.516	/		*p =* 0.345
Coronavirus HKU-1	Positive tests / total tests	3 / 100	3 / 137	3 / 123	0 / 93	9 / 453
	Positivity rate	3.0%	2.2%	2.4%	0.0%	2.0%
	Significance level	*p =* 0.247	*p =* 0.274	*p =* 0.261		*p =* 0.453
Coronavirus NL63	Positive tests / total tests	2 / 100	2 / 137	2 / 123	1 / 93	7 / 453
	Positivity rate	2.0%	1.5%	1.6%	1.1%	1.5%
	Significance level	*p =* 1.000	*p =* 1.000	*p =* 1.000		*p =* 1.000
Coronavirus OC43	Positive tests / total tests	2 / 100	3 / 137	2 / 123	1 / 93	8 / 453
	Positivity rate	2.0%	2.2%	1.6%	1.1%	1.8%
	Significance level	*p =* 1.000	*p =* 0.649	*p =* 1.000		*p =* 0.964
SARS-CoV-2	Positive tests / total tests	/	/	1 / 134	19 / 2564	20 / 2698
	Positivity rate	/	/	0.7%	0.7%	0.7%
	Significance level	/	/	*p =* 1.000		*p =* 1.000
Chlamydophila pneumoniae	Positive tests / total tests	3 / 100	1 / 137	1 / 123	0 / 93	5 / 453
	Positivity rate	3.0%	0.7%	0.8%	0.0%	1.1%
	Significance level	*p =* 0.247	*p =* 1.000	*p =* 1.000		*p =* 0.246
Chlamydophila psittaci	Positive tests / total tests	0 / 100	0 / 137	0 / 123	0 / 93	0 / 453
	Positivity rate	0.0%	0.0%	0.0%	0.0%	0.0%
	Significance level	/	/	/		**/**
Coxiella burnetii	Positive tests / total tests	0 / 100	0 / 137	0 / 123	0 / 93	0 / 453
	Positivity rate	0.0%	0.0%	0.0%	0.0%	0.0%
	Significance level	/	/	/		/
Cytomegalovirus	Positive tests / total tests	27 / 100	40 / 137	31 / 123	19 / 93	117 / 453
	Positivity rate	27.0%	29.2%	25.2%	20.4%	25.8%
	Significance level	*p =* 0.286	*p =* 0.136	*p =* 0.411		*p =* 0.511
Enterovirus D68	Positive tests / total tests	0 / 34	0 / 137	2 / 123	0 / 93	2 / 387
	Positivity rate	0.0%	0.0%	1.6%	0.0%	0.5%
	Significance level	/	/	*p =* 0.507		*p =* 0.326
Herpes simplex virus 1	Positive tests / total tests	4 / 100	8 / 137	6 / 123	3 / 93	21 / 453
	Positivity rate	4.0%	5.8%	4.9%	3.2%	4.6%
	Significance level	*p =* 1.000	*p =* 0.532	*p =* 0.735		*p =* 0.845
Herpes simplex virus 2	Positive tests / total tests	0 / 100	0 / 137	0 / 123	0 / 93	0 / 453
	Positivity rate	0.0%	0.0%	0.0%	0.0%	0.0%
	Significance level	/	/	/		/
Legionella pneumophila	Positive tests / total tests	0 / 100	0 / 137	0 / 123	0 / 93	0 / 453
	Positivity rate	0.0%	0.0%	0.0%	0.0%	0.0%
	Significance level	/	/	/		/
MERS-coronavirus	Positive tests / total tests	0 / 100	0 / 137	0 / 123	0 / 93	0 / 453
	Positivity rate	0.0%	0.0%	0.0%	0.0%	0.0%
	Significance level	/	/	/		/
Mycoplasma pneumoniae	Positive tests / total tests	1 / 100	2 / 137	5 / 123	0 / 93	8 / 453
	Positivity rate	1.0%	1.5%	4.1%	0.0%	1.8%
	Significance level	*p =* 1.000	*p =* 0.516	*p =* 0.072		*p =* 0.171
Parechovirus	Positive tests / total tests	6 / 100	5 / 137	4 / 123	5 / 93	20 / 453
	Positivity rate	6.0%	3.6%	3.3%	5.4%	4.4%
	Significance level	*p =* 0.852	*p =* 0.530	*p =* 0.504		*p =* 0.698
Pneumocystis jirovecii	Positive tests / total tests	8 / 100	4 / 137	12 / 123	7 / 93	31 / 453
	Positivity rate	8.0%	2.9%	9.8%	7.5%	6.8%
	Significance level	*p =* 0.903	*p =* 0.125	*p =* 0.568		*p =* 0.122
SARS-associated coronavirus	Positive tests / total tests	0 / 100	0 / 137	0 / 85	0 / 0	0 / 322
	Positivity rate	0.0%	0.0%	0.0%	0.0%	0.0%
	Significance level	/	/	/		/
Streptococcus pneumoniae	Positive tests / total tests	61 / 100	84 / 137	68 / 123	53 / 93	266 / 453
	Positivity rate	61.0%	61.3%	55.3%	57.0%	58.7%
	Significance level	*p =* 0.572	*p =* 0.513	*p =* 0.803		*p =* 0.731

Statistical differences in occurrence were explored using the Chi-squared or Fisher's exact test. The Fisher's exact test was used when at least one of the values in the 2 x 2 contingency table was smaller than five. Bonferroni correction was applied to compare pathogen occurrence of period 2020-2021 to period 2017-2018, 2018-2019, or 2019-2020 (with significance level provided under each respective column), with an adjusted p-value < 0.01 considered significant (*). For comparison of pathogen occurrence across all four periods (with significance level provided under the column “Total”), a p-value < 0.05 was considered significant (*). Human rhinovirus/enterovirus (†) includes the separate data of rhinovirus and enterovirus until January 2018 as described in Methods.

### Influenza viruses

**Figure 2:**
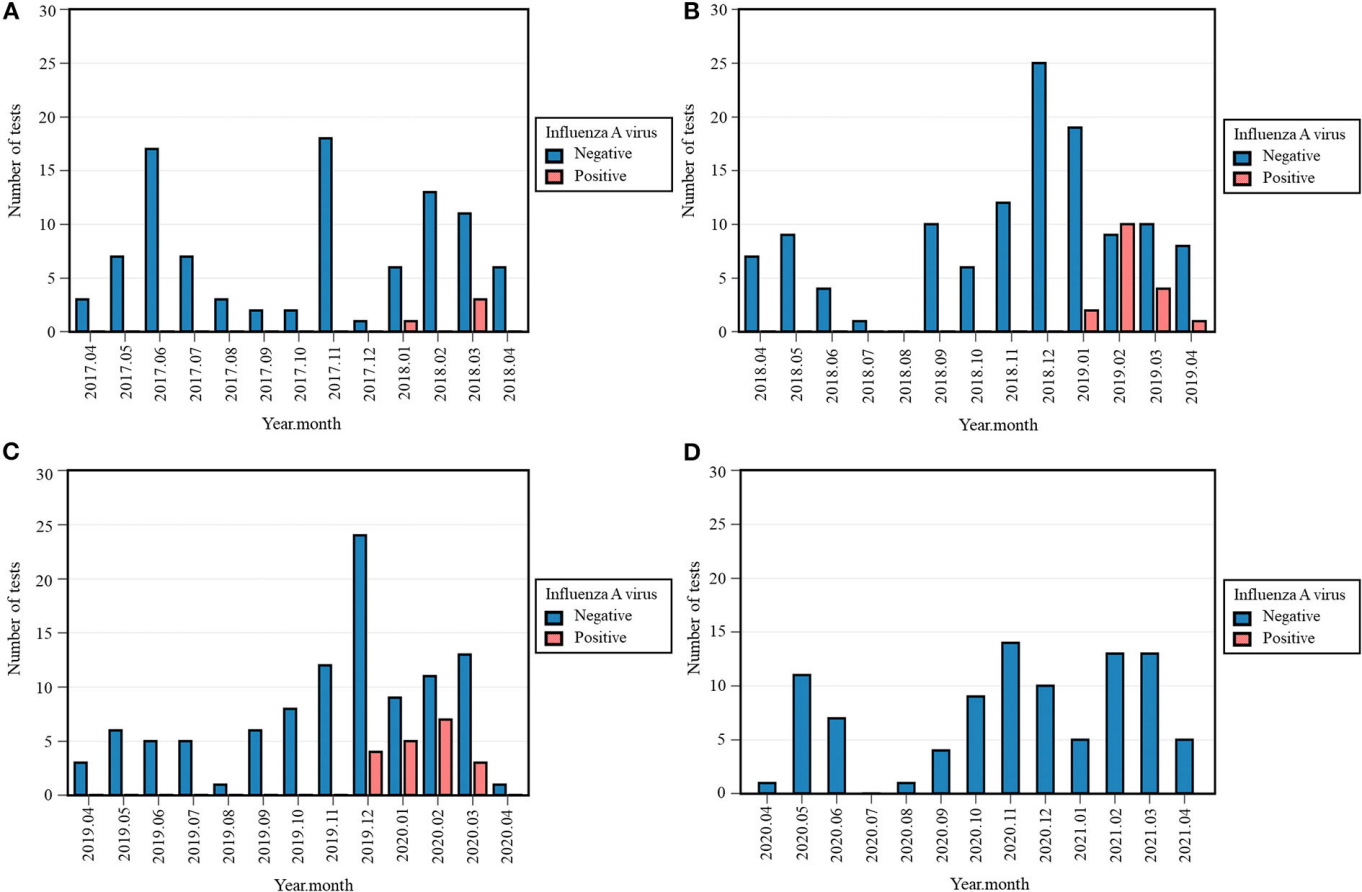
Monthly occurrence of influenza A virus (IAV) per year: 2017-2018 **(A)**, 2018-2019 **(B)**, 2019-2020 **(C)**, 2020-2021 **(D)**. Monthly occurrence was defined as the number of positive tests per month. Each 12-month period started on April 16th and ended on April 15th of the following year.

No cases of IAV were detected in 2020-2021, which contrasts with previous years: four (4%) positive tests in 2017-2018, 17 (12.4%) in 2018-2019, and 19 (15.4%) in 2019-2020 (Table 3, Supplementary Figure S2). The Fisher's exact test across all four periods was significant (*p* < 0.001). Positivity rate in 2020-2021 was significantly lower than 2018-2019 (*p* < 0.001) and 2019-2020 (*p* < 0.001). IAV was observed exclusively between December and April, with its last case detected in March 2020 (Figure 2).

As for IBV, four (4%) and two (1.6%) positives were detected from February to March 2018 and from December 2019 to January 2020, respectively (Table 3). The Fisher's exact test across all four periods was significant (*p* = 0.020), although the differences in occurrence between 2020-2021 and the previous periods were non-significant.

### Respiratory syncytial virus

**Figure 3:**
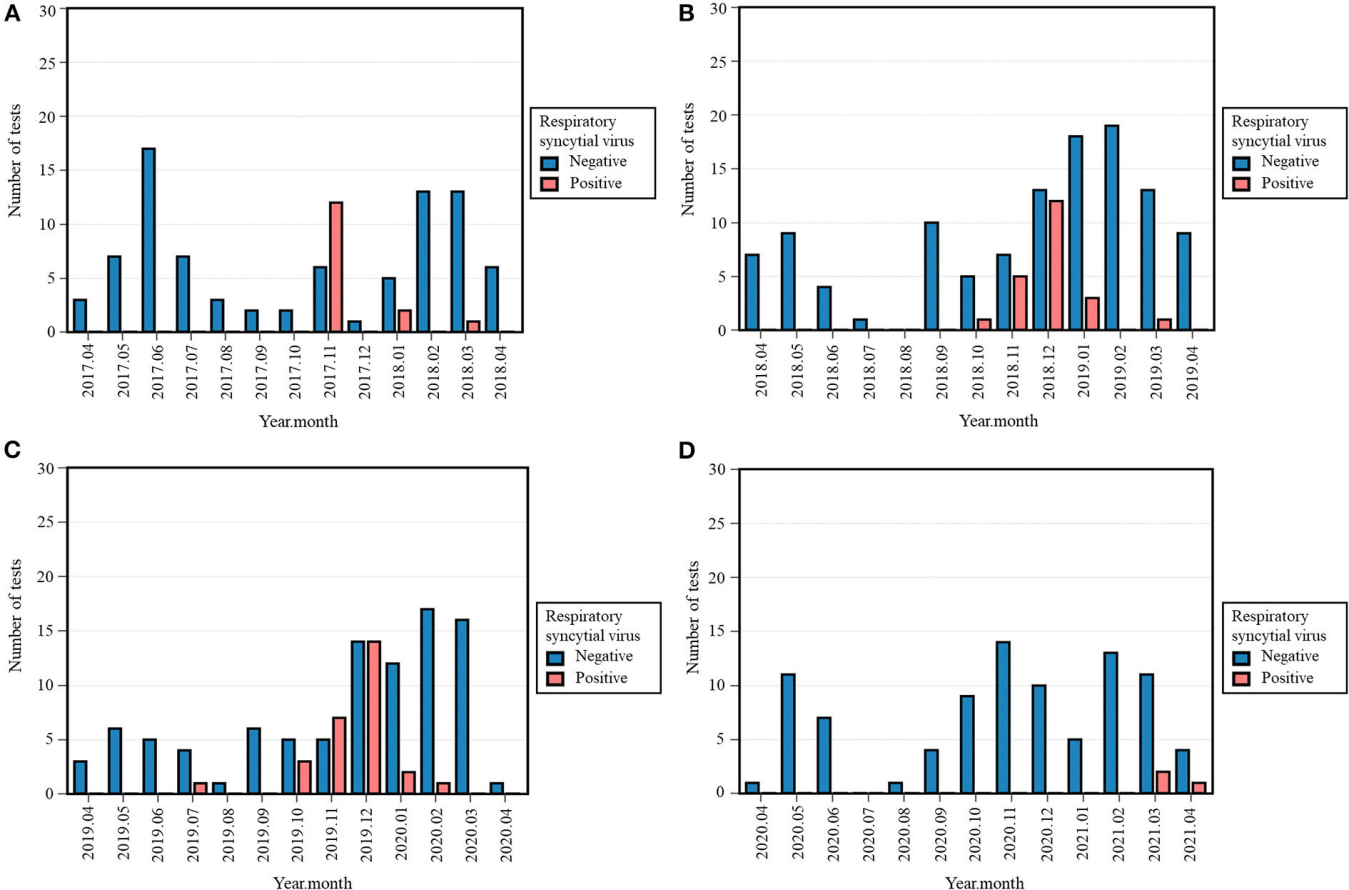
Monthly occurrence of respiratory syncytial virus (RSV) per year: 2017-2018 **(A)**, 2018-2019 **(B)**, 2019-2020 **(C)**, 2020-2021 **(D)**. Monthly occurrence was defined as the number of positive tests per month. Each 12-month period started on April 16th and ended on April 15th of the following year.

With only three (3.2%) cases of RSV in 2020-2021, its occurrence decreased compared to 15 (15.0%) positive tests in 2017-2018, 22 (16.1%) in 2018-2019, and 28 (22.8%) in 2019-2020 (Table 3, Supplementary Figure S3). The Fisher's exact test across all four periods was significant (*p* < 0.001). Positivity rate of RSV in 2020-2021 was significantly lower than 2017-2018 (*p* = 0.006), 2018-2019 (*p* = 0.002), and 2019-2020 (*p* < 0.001). Remarkably, its characteristic winter peak was absent in 2020-2021, as the positive tests were observed later, i.e., March and April 2021 (Figure 3).

### Parainfluenza viruses

**Figure 4:**
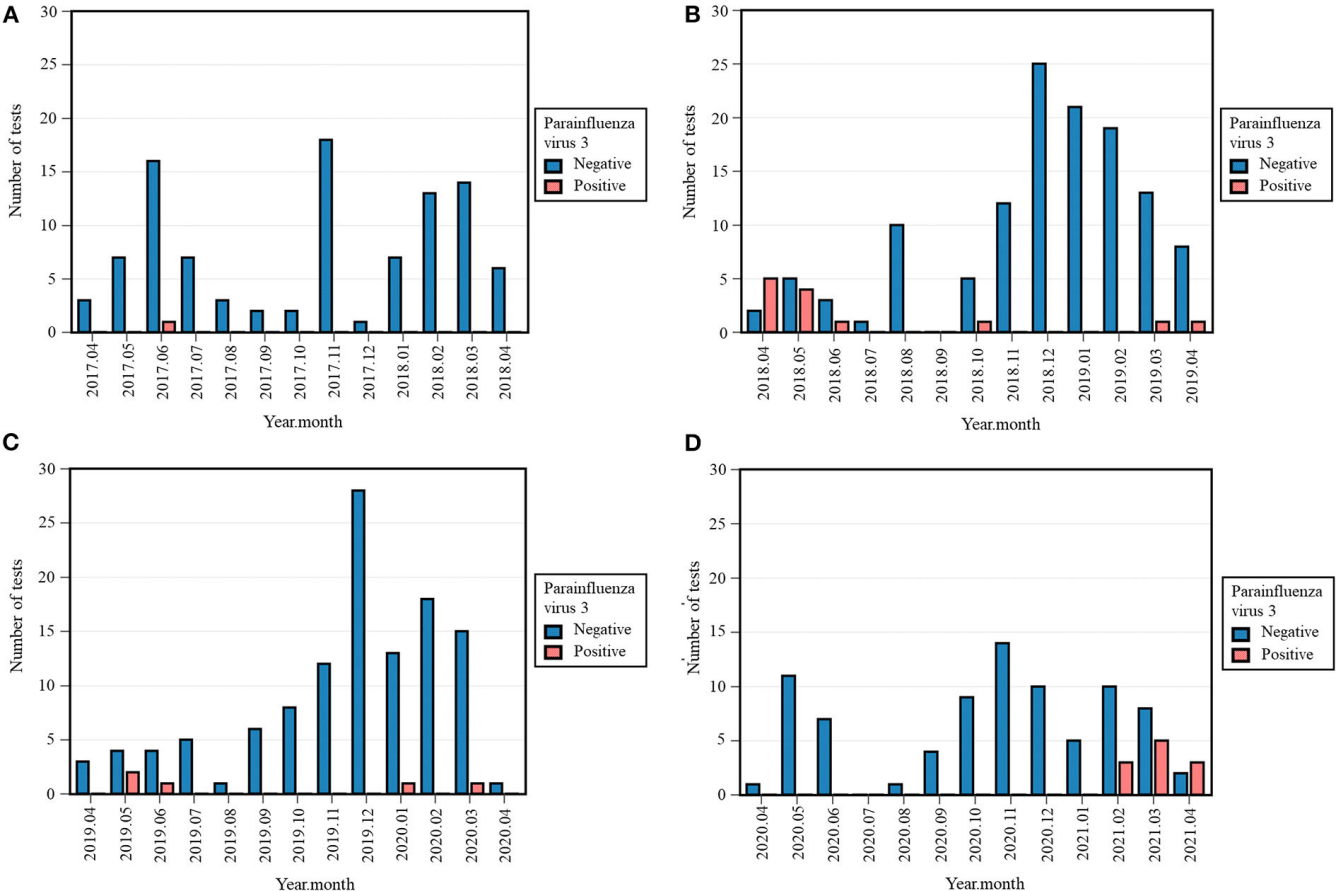
Monthly occurrence of parainfluenza virus 3 (PIV-3) per year: 2017–2018 **(A)**, 2018-2019 **(B)**, 2019–2020 **(C)**, 2020-2021 **(D)**. Monthly occurrence was defined as the number of positive tests per month. Each 12-month period started on April 16th and ended on April 15th of the following year.

Zero (0%) cases of PIV-1 were detected in 2018-2019 and 2020-2021, in contrast with two (2.0%) in 2017-2018 and five (4.1%) in 2019-2020 (Table 3). The Fisher's exact test across all four periods was significant (*p* = 0.021), while the differences in occurrence between 2020-2021 and previous periods were not. No cases of PIV-2, and PIV-4 were detected in 2020-2021. As the absolute number of positive tests in the previous years was equally low, the difference between 2020-2021 and the previous periods were non-significant (Table 3). However, eleven (11.8%) tests were positive for PIV-3 in 2020-2021, compared to one (1%) in 2017-2018, 13 (9.5%) in 2018-2019, and five (4.1%) in 2019-2020 (Table 3, Supplementary Figure S4). The Fisher's exact test across all four periods was significant (*p* = 0.004) and the positivity rate of PIV-3 in 2020-2021 was only significantly higher than 2017-2018 (*p* = 0.002, Fisher's exact). After Bonferroni correction, the difference in occurrence between 2019-2020 and 2020-2021 was considered non-significant (*p* = 0.031, Chi-squared). PIV-3 remained absent from April 2020 until January 2021, whereas no clear seasonal pattern could be distinguished during other years. From February 2021 onwards, a rising trend was seen as represented in Figure 4.

### Adenovirus

In 2017-2018, 26 (26%) cases of ADV were detected, compared to 29 (21.2%) in 2018-2019, 16 (13%) in 2019-2020, and 15 (16.1%) in 2020-2021 (Table 3). No significant differences in occurrence of ADV were observed across the four periods. ADV circulated throughout the year during the study period, except for a period of zero positives between May and October 2018.

### Bocavirus

Ten (10.0%) samples tested positive for bocavirus in 2017-2018 (Table 3). Additionally, 13 (9.5%) samples were positive in 2018-2019, and six (4.9%) in 2019-2020. Compared to nine (9.7%) positives in 2020-2021, the differences in occurrence are non-significant. Except for two cases in February and March 2020, no cases were detected between July 2019 and October 2020.

### Human metapneumovirus

Regarding hMPV, five (5.0%) cases were detected in 2017-2018, compared to nine (6.6%) in 2018-2019, three (2.4%) in 2019-2020, and four (4.3%) in 2020-2021 (Table 3). The differences in occurrence across the four periods were not significant. Its appearance between December and April was preserved during all four periods.

### Human rhinovirus/enterovirus

HRV/ENT propagated throughout the year with 54 (32.5%) positive samples in 2017-2018, 56 (40.9%) in 2018-2019, 59 (48.0%) in 2019-2020 and 44 (47.3%) in 2020-2021 (Table 3). The Chi-squared test across the four periods was not statistically significant.

### Common coronaviruses

During the study period, only two (1.5%) cases of coronavirus 229E were detected in 2018-2019 (Table 3). In contrast to zero positives in 2020-2021, three tests were positive for coronavirus HKU-1 in 2017-2018 (3.0%), 2018-2019 (2.2%), and 2019-2020 (2.4%) (Table 3). Compared to one (1.1%) case in 2020-2021, two cases of coronavirus NL63 were identified in 2017-2018 (2.0%), 2018-2019 (1.5%), and 2019-2020 (1.6%) (Table 3). A single (1.1%) sample tested positive for coronavirus OC43 in 2020-2021, compared to two in 2017-2018 (2.0%) and 2019-2020 (1.6%), and three (2.2%) in 2018-2019 (Table 3). For these common coronaviruses (229E, HKU-1, NL63, OC43), no statistically significant differences in occurrence were observed across the four periods (Table 3).

### SARS-CoV-2

With the first case detected in March 2020, 20 (0.7%) tests were positive for SARS-CoV-2 during the investigated study period (Table 3). The highest peak observed consisted of five (2.9%) positives in November 2020 (Supplementary Figure S5).

### Other respiratory pathogens

The absolute number of positive tests and positivity rate of other respiratory pathogens included in the respiratory panel are listed in Table 3. None showed a significant difference in occurrence between the 2020-2021 period and the previous periods.

### Pathogen distribution by age

The distribution of respiratory pathogens during the study period for the different ages are listed in Supplementary Table S1. The proportion of IAV infections was highest in the age groups of 2 to 5 years old (6.8–8.2% of all positive tests for each age group). A similar pattern was observed for IBV in the age categories of 3 and 4 years old, with 2.1 and 3.4% of all positive tests respectively. With RSV, the ages of 0 (8.6% of all positive tests) and 1 years old (7.3% of all positive tests) were most commonly affected, compared to other age categories. Albeit that the number of positive tests for parainfluenza virus were low, the ages of 1 (4.3% of all positive tests) and 2 (3.7% of all positive tests) had the highest proportions of PIV-3 infections. For further details on the remaining pathogens, we refer to Supplementary Table S1.

## Discussion

### Respiratory infection epidemiology

This retrospective study investigated the collateral effect of the NPIs adopted to reduce the spread of SARS-CoV-2 on the occurrence of viral respiratory pathogens in young children. As for social restrictions in Belgium, the first lockdown lasted from March until beginning of May 2020 (39–42). Except for children of essential workers, schools were closed during this period. Subsequently, from October 2022, new restrictions were gradually implemented (43, 44), until gradual facilitation as of early 2021 (45, 46). See Supplementary Figure S6 for a timeline as graphical representation of a selection of NPIs, of relevance for this paper, implemented in Belgium. The figure is based on data and definitions from the European Centre for Disease Prevention and Control, to allow comparison with other countries (47). We compared the occurrence of viral respiratory pathogens in the period of April 2020 to April 2021 with three previous 12-month periods and report two major findings.

First, we observed a complete absence of IAV and IBV from March 2020 and January 2020 respectively, until the end of the study period. These findings are similar to those reported by another retrospective analysis of the Wuhan University Hospital, investigating the occurrence of influenza in children presenting between September and December from 2018 until 2020 (26). They reported a significantly lower positivity rate in 2020 (4.3%), compared to 2018 (14.0%) and 2019 (28.7%) (26). Likewise, an Italian tertiary hospital retrospectively compared the three winter seasons (September to February) from 2018 to 2021 (35). They did not detect any influenza virus in patients aged 0–18 years old during the last season, which contrasted with 2018-2019 (*n* = 240) and 2019-2020 (*n* = 354) (35). In that study, only five cases of RSV were detected in 2020-2021, compared to 2018-2019 (*n* = 726) and 2019-2020 (*n* = 689) (35). A Finnish study reported a shorter influenza and RSV season in early 2020 with a steeper decline from peak to zero cases than in the four previous years (8 vs. 13–20 weeks) (25). In the Southern Hemisphere as well, a significant reduction in detections of influenza (99.4%) and RSV (98.0%) was reported during the 2020 Australian winter compared to 2012-2019 (15). Our results are consistent with observations of the Belgian sentinel laboratories, reporting a reappearance of influenza virus only in December 2021 (48).

Second, we observed an absence of RSV from March 2020 onwards. Without its characteristic winter peak in November-December, RSV emerged in March 2021. The Belgian sentinel laboratories reported a more than 99% reduction in nationally diagnosed RSV infections during the 2020-2021 winter (29). In December 2020, the authors warned for a delayed RSV peak when the adopted public health measures would be lifted gradually (29). Their forewarning has come true as the sentinel laboratories noted a rising trend starting in February 2021 with a smaller peak in March 2021 and a larger peak in May 2021 (48). From June 2021 onwards, a downward trend was observed until resurgence in October 2021 (48). Countries all over the globe have reported a delayed start of the RSV epidemic, including France (3-4 months after usual onset), South Africa (5 months), the United States and Australia (6 months), the Netherlands and Japan (7 months), and New Zealand (12 months) (32, 33). This unusual RSV occurrence from early to mid 2021, separate from its usual seasonality, has been described in literature as a global phenomenon (49–52).

Besides the two major findings discussed above, some additional results were of interest. We recognized a similar trend of PIV-3 in line with RSV, characterized by no cases from April 2020 to January 2021 and delayed emergence in February 2021. The annual positivity rate in 2020-2021, which at first sight might seem in conflict with an effect of NPIs, is solely attributed to the eleven positive tests which were detected in the last 2.5 months of the 2020-2021 period. Without differentiation between serotypes, the Belgian sentinel laboratories observed a decrease in occurrence of PIV starting March 2020 (48). In their report, the weekly number of positive cases remained low until a resurgence started in January 2021, peaking in March 2021 (48). The other respiratory viruses investigated in this study showed no statistically significant differences in occurrence in 2020-2021, compared to the three previous periods. ADV circulated throughout the year, in accordance with observations of the Belgian sentinel laboratories (48). The degree of impact from NPIs on viral epidemiology might in part be attributed to differences in structural pathogen characteristics, such as the presence (enveloped viruses) or absence (non-enveloped viruses) of a lipid envelope (53). Notably, enveloped viruses (such as influenza viruses, RSV, PIV, and coronaviruses) are vulnerable to inactivation by alcohol-containing hand sanitizers and desinfectants (53). In contrast, non-enveloped viruses (such as human RV, ADV, and bocavirus) have relatively low sensitivity to alcohol-based inactivation (53). Comparing the timing of the implemented NPIs with our results, the absence of the affected respiratory viruses from March 2020 (RSV) and April 2020 (IAV, PIV-3) onwards can be associated with the first NPIs (including stay-at-home order, closure of schools and non-essential shops) being implemented as of March 2020. Subsequently, the resurgence of these viral pathogens starting February 2021 (PIV-3) and March 2021 (RSV) might be explained by the gradual relaxation of social measures (such as private and gatherings) as of early 2021.

In a hospital-based observational study from Taiwan, Chen et al. compared January-December 2019 to January-May 2020 and reported they did not observe epidemiological impact of NPIs on non-enveloped viruses (54). Instead, they noted an increase in HRV/ENT positivity rate in pediatric patients (54). A Canadian observational study compared the 2020-2021 season to five pre-pandemic respiratory virus seasons (2014-2019) and observed a dramatically lower incidence of seasonal respiratory viruses (including IAV, IBV, RSV, PIV, hMPV), except for ADV and HRV/ENT (27). The abovementioned Italian retrospective study reported a reduction in occurrence of RV in 2020-2021, albeit to a lesser extent than influenza viruses and RSV (35). Likewise, Kume et al. reported an overall decrease in detected cases of RV, ADV, and bocavirus in hospitalized children after implementation of NPIs against SARS-CoV-2, but that these non-enveloped viruses were nonetheless consistently detected (55).

### Strengths and limitations

We were able to gather an extensive dataset for this single-center retrospective study, from a large university hospital. As our dataset concerned young children presenting at this hospital, our results represent the local epidemiology. Although our results are in line with reports in literature, it is of relevance to analyze data on a regional scale. Especially since regional or international differences in degree and timing of NPIs might impede reliable comparison.

Nevertheless, we are aware that this dataset gives a biased view on the circulation of respiratory pathogens in the pediatric population. First, children with a milder clinical course of disease may not need medical attention and therefore, the causative agent will never be identified and documented. Second, compared to a multi-center analysis, a small difference in the data of this single hospital center might have a larger effect on the results. Third, one must acknowledge the subjective threshold for physicians of taking a respiratory sample in a child who does not require hospitalization. Since the cost of the analysis is often at the expense of the patient, physicians might be reluctant to systematically order a respiratory panel. Hence, the dataset concerned patients for whom it was expected to offer added diagnostic value. As mentioned above, the data concerned 453 respiratory panels of 411 unique patients. Based on this retrospective dataset, no consideration of underlying medical conditions of the included cases was possible. Lastly, the impact of the COVID-19 pandemic on the healthcare-seeking behavior of the population must not be taken lightly and might introduce bias. We are aware that differences in testing strategy and abovementioned remarks might influence the results. Consequently, we consider the results to be mainly explorative.

### Future perspectives

The changes in epidemiology demonstrate that NPI can impact occurrence of viral respiratory infections in young children. A question remains to what extent NPIs such as face masks and lockdowns will be considered in managing any future epi- and pandemics, such as influenza (56, 57). Better understanding could lead to specific interventions to reduce the burden of these viral infections.

As social interaction in the absence of public health measures increases, healthcare providers should be wary of co-circulation of SARS-CoV-2 among other respiratory pathogens. The implications of coinfection with viruses and atypical bacteria on clinical features, laboratory and radiological examinations, treatments and outcomes are still unclear (58). Given that young children have the highest frequency of coinfections (notably with RSV and PIV or ADV), one must anticipate a future increment of coinfections with common respiratory viruses and SARS-CoV-2 (11, 59, 60).

Since this is a Belgian single-center analysis until April 2021, we provide a piece of the global picture of viral respiratory infection epidemiology during the COVID-19 pandemic. It is important to monitor this evolution through epidemiological surveillance, keeping in mind differences in local NPIs to allow reliable comparison and interpretation. Given the pandemic is still ongoing at the time of writing of this manuscript, we strongly encourage the repetition of this analysis in a few years, more in-depth and on a multi-center scale.

## Data availability statement

The raw data supporting the conclusions of this article will be made available by the authors, without undue reservation.

## Ethics statement

The studies involving human participants were reviewed and approved by Educational-Support Committee of the Research Ethics Committee UZ/KU Leuven. Written informed consent from the participants' legal guardian/next of kin was not required to participate in this study in accordance with the national legislation and the institutional requirements.

## Author contributions

DS: study conception, study design, data interpretation, and manuscript draft. AS: study conception, study design, data acquisition, data interpretation, and manuscript review. FV: study conception, data interpretation, and manuscript review. KL and AD: data interpretation and manuscript review. All authors have reviewed the final manuscript and have given approval for publishing.

## Conflict of interest

The authors declare that the research was conducted in the absence of any commercial or financial relationships that could be construed as a potential conflict of interest.

## Publisher's note

All claims expressed in this article are solely those of the authors and do not necessarily represent those of their affiliated organizations, or those of the publisher, the editors and the reviewers. Any product that may be evaluated in this article, or claim that may be made by its manufacturer, is not guaranteed or endorsed by the publisher.

## Abbreviations

ADV: Adenovirus
COVID-19: Coronavirus disease of 2019
EV: Enterovirus
hMPV: Human metapneumovirus
HRV/ENT: Human rhinovirus/enterovirus
IAV: Influenza A virus
IBV: Influenza B virus
NPIs: Non-pharmaceutical interventions
PIV: Parainfluenza virus
RSV: Respiratory syncytial virus
RV: Rhinovirus
SARS-CoV-2: Severe Acute Respiratory Syndrome coronavirus 2.

## References

[1] Imai N Gaythorpe KAM Abbott S Bhatia S van Elsland S Prem K. Adoption and impact of non-pharmaceutical interventions for COVID-19. Wellcome Open Res. (2020) 5:59. 10.12688/wellcomeopenres.15808.1 32529040 PMC7255913

[2] Huang QS Wood T Jelley L Jennings T Jefferies S Daniells K. Impact of the COVID-19 nonpharmaceutical interventions on influenza and other respiratory viral infections in New Zealand. Nat Commun. (2021) 12:1001. 10.1038/s41467-021-21157-9 33579926 PMC7881137

[3] Dolk FCK de Boer PT Nagy L Donker GA Meijer A Postma MJ. Consultations for influenza-like illness in primary care in the netherlands: a regression approach. Value Health. (2021) 24:11–8. 10.1016/j.jval.2020.10.013 33431142

[4] GBD 2016 Lower Respiratory Infections Collaborators. Estimates of the global, regional, and national morbidity, mortality, and aetiologies of lower respiratory infections in 195 countries, 1990-2016: a systematic analysis for the Global Burden of Disease Study 2016. Lancet Infect Dis. (2018) 18:1191–210. 10.1016/S1473-3099(18)30310-4 30243584 PMC6202443

[5] Wang X Li Y Mei X Bushe E Campbell H Nair H. Global hospital admissions and in-hospital mortality associated with all-cause and virus-specific acute lower respiratory infections in children and adolescents aged 5-19 years between 1995 and 2019: a systematic review and modelling study. BMJ Glob Health. (2021) 6:e006014. 10.1136/bmjgh-2021-006014 34261758 PMC8281096

[6] Wang X Li Y O'Brien KL Mahdi SA Widdowson MA Byass P. Global burden of respiratory infections associated with seasonal influenza in children under 5 years in 2018: a systematic review and modelling study. Lancet Glob Health. (2020) 8:e497–510. 10.1016/S2214-109X(19)30545-5 32087815 PMC7083228

[7] Li Y Wang X Blau DM Caballero MT Feikin DR Gill CJ. Global, regional, and national disease burden estimates of acute lower respiratory infections due to respiratory syncytial virus in children younger than 5 years in 2019: a systematic analysis. Lancet. (2022) 399:2047–64. 10.1016/S0140-6736(22)00478-0 35598608 PMC7613574

[8] Wang X Li Y Deloria-Knoll M Mahdi SA Cohen C Arguelles VL. Global burden of acute lower respiratory infection associated with human parainfluenza virus in children younger than 5 years for 2018: a systematic review and meta-analysis. Lancet Glob Health. (2021) 9:e1077–87. 10.1016/S2214-109X(21)00218-7 34166626 PMC8298256

[9] Wang X Li Y Deloria-Knoll M Mahdi SA Cohen C Ali A. Global burden of acute lower respiratory infection associated with human metapneumovirus in children under 5 years in 2018: a systematic review and modelling study. Lancet Glob Health. (2021) 9:e33–43. 10.1016/S2214-109X(20)30393-4 33248481 PMC7783516

[10] Moriyama M Hugentobler WJ Iwasaki A. Seasonality of Respiratory Viral Infections. Annual Rev Virol. (2020) 7:83–101. 10.1146/annurev-virology-012420-022445 32196426

[11] Ramaekers K Keyaerts E Rector A Borremans A Beuselinck K Lagrou K. Prevalence and seasonality of six respiratory viruses during five consecutive epidemic seasons in Belgium. J Clinical Virol. (2017) 94:72–8. 10.1016/j.jcv.2017.07.011 28772168

[12] Nair H Nokes DJ Gessner BD Dherani M Madhi SA Singleton RJ. Global burden of acute lower respiratory infections due to respiratory syncytial virus in young children: a systematic review and meta-analysis. Lancet. (2010) 375:1545–55. 10.1016/S0140-6736(10)60206-1 20399493 PMC2864404

[13] Stein RT Bont LJ Zar H Polack FP Park C Claxton A. Respiratory syncytial virus hospitalization and mortality: Systematic review and meta-analysis. Pediatr Pulmonol. (2017) 52:556–69. 10.1002/ppul.23570 27740723 PMC5396299

[14] Mehta NS Mytton OT Mullins EWS Fowler TA Falconer CL Murphy OB. SARS-CoV-2 (COVID-19): what do we know about children? a systematic review. Clin Infect Diseases. (2020) 71:2469–79. 10.1093/cid/ciaa556 32392337 PMC7239259

[15] Zimmermann P Curtis N. COVID-19 in children, pregnancy and neonates: a review of epidemiologic and clinical features. Pediatr Infecti Dis J. (2020) 39:469–77. 10.1097/INF.0000000000002700 32398569 PMC7363381

[16] Bhuiyan MU Stiboy E. Hassan MdZ, Chan M, Islam MdS, Haider N, et al. Epidemiology of COVID-19 infection in young children under five years: a systematic review and meta-analysis. Vaccine. (2021) 39:667–77. 10.1016/j.vaccine.2020.11.078 33342635 PMC7833125

[17] Melidou A Pereyaslov D Hungnes O Prosenc K Alm E Adlhoch C. Virological surveillance of influenza viruses in the WHO European Region in 2019/20—impact of the COVID-19 pandemic. Euro Surveill. (2020) 25:2001822. 10.2807/1560-7917.ES.2020.25.46.2001822 33213683 PMC7678039

[18] Olsen SJ Azziz-Baumgartner E Budd AP Brammer L Sullivan S Pineda RF. Decreased influenza activity during the COVID-19 Pandemic—United States, Australia, Chile, and South Africa, 2020. MMWR Morb Mortal Wkly Rep. (2020) 69:1305–9. 10.15585/mmwr.mm6937a6 32941415 PMC7498167

[19] Lee H Lee H Song K-H Kim ES Park JS Jung J. Impact of Public Health Interventions on Seasonal Influenza Activity During the COVID-19 Outbreak in Korea. Clin Infect Dis. (2021) 73:e132–40. 10.1093/cid/ciaa672 32472687 PMC7314207

[20] Soo RJJ Chiew CJ Ma S Pung R Lee V. Decreased influenza incidence under COVID-19 control measures, Singapore. Emerg Infect Dis. (2020) 26:1933–5. 10.3201/eid2608.201229 32339092 PMC7392467

[21] Kuo S-C Shih S-M Chien L-H Hsiung CA. Collateral benefit of COVID-19 control measures on influenza activity, Taiwan. Emerg Infect Dis. (2020) 26:1928–30. 10.3201/eid2608.201192 32339091 PMC7392415

[22] Lipsitch M Swerdlow DL Finelli L. Defining the Epidemiology of Covid-19—Studies Needed. NEJM. (2020) 382:1194–6. 10.1056/NEJMp2002125 32074416

[23] Fricke LM Glöckner S Dreier M Lange B. Impact of non-pharmaceutical interventions targeted at COVID-19 pandemic on influenza burden—a systematic review. J Infection. (2021) 82:1–35. 10.1016/j.jinf.2020.11.039 33278399 PMC9183207

[24] Yeoh DK Foley DA Minney-Smith CA Martin AC Mace AO Sikazwe CT. Impact of Coronavirus Disease 2019 public health measures on detections of influenza and respiratory syncytial virus in children during the 2020 Australian Winter. Clin Infect Dis. (2021) 72:2199–202. 10.1093/cid/ciaa1475 32986804 PMC7543326

[25] Kuitunen I Artama M Mäkelä L Backman K Heiskanen-Kosma T Renko M. Effect of social distancing due to the COVID-19 pandemic on the incidence of viral respiratory tract infections in children in Finland During early 2020. Pediatr Infect Dis J. (2020) 39:e423–7. 10.1097/INF.0000000000002845 32773660

[26] Yu X Xu C Huang W Xu X Xie W Long X. The incidence of influenza in children was decreased in the first flu season after COVID-19 pandemic in Wuhan. J Infect Public Health. (2021) 14:1279–81. 10.1016/j.jiph.2021.08.027 34500253 PMC8393498

[27] Groves HE Piché-Renaud P-P Peci A Farrar DS Buckrell S Bancej C. The impact of the COVID-19 pandemic on influenza, respiratory syncytial virus, and other seasonal respiratory virus circulation in Canada: a population-based study. Lancet Reg Health Am. (2021) 1:100015. 10.1016/j.lana.2021.100015 34386788 PMC8285668

[28] Hatoun J Correa ET Donahue SMA Vernacchio L. Social Distancing for COVID-19 and diagnoses of other infectious diseases in children. Pediatrics. (2020) 146:e2020006460. 10.1542/peds.2020-006460 32879032

[29] van Brusselen D de Troeyer K. ter Haar E, vander Auwera A, Poschet K, van Nuijs S, et al. Bronchiolitis in COVID-19 times: a nearly absent disease? Eur J Pediatr. (2021) 180:1969–73. 10.1007/s00431-021-03968-6 33517482 PMC7847293

[30] Avadhanula V Piedra PA. The prevention of common respiratory virus epidemics in 2020-21 during the severe acute respiratory syndrome Coronavirus 2 (SARS-CoV-2) Pandemic: an unexpected benefit of the implementation of public health measures. Lancet Reg Health Am. (2021) 2:100043. 10.1016/j.lana.2021.100043 34430955 PMC8377442

[31] Zhang Y Qiao L Yao J Yu N Mu X Huang S. Epidemiological and clinical characteristics of respiratory viruses in 4403 pediatric patients from multiple hospitals in Guangdong, China. BMC Pediatr. (2021) 21:284. 10.1186/s12887-021-02759-0 34140022 PMC8212487

[32] Williams TC Sinha I Barr IG Zambon M. Transmission of paediatric respiratory syncytial virus and influenza in the wake of the COVID-19 pandemic. Eurosurveillance. (2021) 26:186. 10.2807/1560-7917.ES.2021.26.29.2100186 34296673 PMC8299749

[33] van Summeren J Meijer A Aspelund G Casalegno JS Erna G Hoang U. Low levels of respiratory syncytial virus activity in Europe during the 2020/21 season: what can we expect in the coming summer and autumn/winter? Eurosurveillance. (2021) 26:639. 10.2807/1560-7917.ES.2021.26.29.2100639 34296672 PMC8299745

[34] Lumley SF Richens N Lees E Cregan J Kalimeris E Oakley S. Changes in paediatric respiratory infections at a UK teaching hospital 2016–2021; impact of the SARS-CoV-2 pandemic. J Infect. (2022) 84:40–7. 10.1016/j.jinf.2021.10.022 34757137 PMC8591975

[35] Vittucci AC Piccioni L Coltella L Ciarlitto C Antilici L Bozzola E. The Disappearance of Respiratory Viruses in Children during the COVID-19 Pandemic. Int J Environ Res Public Health. (2021) 18:9550. 10.3390/ijerph18189550 34574472 PMC8467075

[36] Kamper-Jørgensen M Benn CS Simonsen J Thrane N Wohlfahrt J. Clustering of acute respiratory infection hospitalizations in childcare facilities. Acta Paediatr. (2010) 99:877–82. 10.1111/j.1651-2227.2010.01712.x 20178520

[37] The University Hospitals Leuven. Labogids Respiratoir Panel. Leuven (Belgium): The University Hospitals. Available online at: https://laboboeken.nexuzhealth.com/pboek/internet/GHB/6855 (accessed August 5, 2022).

[38] The University Hospitals Leuven. Wekelijkse detectieresultaten respiratoire pathogenen in UZ Leuven. Leuven (Belgium): The University Hospitals. Available online at: https://www.uzleuven.be/nl/laboratoriumgeneeskunde/wekelijkse-detectieresultaten-respiratoire-pathogenen (accessed August 5, 2022).

[39] de Crem P. 13 MAART 2020–Ministerieel besluit houdende dringende maatregelen om de verspreiding van het coronavirus COVID-19 te beperken. Brussels (Belgium): Belgisch Staatsblad. (2020). Available online at: https://www.ejustice.just.fgov.be/eli/besluit/2020/03/13/2020030303/staatsblad (accessed August 5, 2022).

[40] de Crem P. 18 MAART 2020. - Ministerieel besluit houdende dringende maatregelen om de verspreiding van het coronavirus COVID-19 te beperken. Brussels (Belgium): Belgisch Staatsblad. (2020). Available online at: https://www.ejustice.just.fgov.be/eli/besluit/2020/03/18/2020030331/staatsblad (accessed August 5, 2022).

[41] de, Crem P,. 30 MEI 2020– Ministerieel besluit houdende wijziging van het ministerieel besluit van 23 maart 2020 houdende dringende maatregelen om de verspreiding van het coronavirus COVID-19 te beperken. Brussels (Belgium): Belgisch Staatsblad. 2020 May 30. Available online at: http://www.ejustice.just.fgov.be/eli/besluit/2020/05/30/2020030965/staatsblad (accessed August 5, 2022).

[42] de, Crem P,. 5 JUNI 2020. - Ministerieel besluit houdende wijziging van het ministerieel besluit van 23 maart 2020 houdende dringende maatregelen om de verspreiding van het coronavirus COVID-19 te beperken. Brussels (Belgium): Belgisch Staatsblad. 2020 Jun 5. Available online at: http://www.ejustice.just.fgov.be/eli/besluit/2020/06/05/2020010398/staatsblad (accessed August 5, 2022).

[43] Verlinden, A,. 28 OKTOBER 2020. - Ministerieel besluit houdende dringende maatregelen om de verspreiding van het coronavirus COVID-19 te beperken. Brussels (Belgium): Belgisch Staatsblad. 2020 Okt 28. Available online at: http://www.ejustice.just.fgov.be/eli/besluit/2020/10/28/2020010455/staatsblad (accessed August 5, 2022).

[44] Verlinden, A,. 6 FEBRUARI 2021. - Ministerieel besluit houdende wijziging van het ministerieel besluit van 28 oktober 2020 houdende dringende maatregelen om de verspreiding van het coronavirus COVID-19 te beperken. Brussels (Belgium): Belgisch Staatsblad. 2021 Feb 7. Available online at: http://www.ejustice.just.fgov.be/eli/besluit/2021/02/06/2021030266/staatsblad (accessed August 5, 2022).

[45] Verlinden, A,. 7 MEI 2021. - Ministerieel besluit houdende wijziging van het ministerieel besluit van 28 oktober 2020 houdende dringende maatregelen om de verspreiding van het coronavirus COVID-19 te beperken. Brussels (Belgium): Belgisch Staatsblad. 2021 May 7. Available online at: http://www.ejustice.just.fgov.be/eli/besluit/2021/05/07/2021031513/staatsblad (accessed August 5, 2022).

[46] Verlinden A. 23 JUNI 2021. - Ministerieel besluit houdende wijziging van het ministerieel besluit van 28 oktober 2020 houdende dringende maatregelen om de verspreiding van het coronavirus COVID-19 te beperken. Brussels (Belgium): Belgisch Staatsblad. (2021). Available online at: http://www.ejustice.just.fgov.be/eli/besluit/2021/06/23/2021010037/staatsblad (accessed August 5, 2022).

[47] European Centre for Disease Prevention Control. Data on Country Response Measures to COVID-19. Solna (Sweden): European Centre for Disease Prevention and Control. 2022 May. Available online at: https://www.ecdc.europa.eu/en/publications-data/download-data-response-measures-covid-19 (accessed May 24, 2022)

[48] Sciensano. Weekly Bulletin Respiratory Infections, Bulletin Hebdomadaire Infections Respiratoires, Weekly Bulletin Respiratory Infections. Brussels (Belgium): Sciensano. (2022). Available online at: https://epidemio.wiv-isp.be/ID/diseases/SiteAssets/Pages/Influenza/WeeklyBulletinRespiratoryInfections.pdf (accessed August 5, 2022).

[49] Jia R Lu L Su L Lin Z Gao D Lv H. Resurgence of respiratory syncytial virus infection during COVID-19 pandemic among children in Shanghai, China. Front Microbiol. (2022) 13:938372. 10.3389/fmicb.2022.938372 35875547 PMC9298468

[50] Ujiie M Tsuzuki S Nakamoto T Iwamoto N. Resurgence of respiratory syncytial virus infections during COVID-19 pandemic, Tokyo, Japan. Emerg Infect Dis. (2021) 27:2969–70. 10.3201/eid2711.211565 34388086 PMC8544984

[51] Anglemyer A Rutherford G Walls T Maldonado Y. Unusual Interseasonal RSV Activity in the Southern and Northern Hemispheres. J Infect Dis. (2022) 225:1680–82. 10.1093/infdis/jiab620 34935959

[52] Agha R Avner JR. Delayed seasonal RSV surge observed during the COVID-19 pandemic. Pediatrics. (2021) 148:e2021052089. 10.1542/peds.2021-052089 34108234

[53] Golin AP Choi D Ghahary A. Hand sanitizers: A review of ingredients, mechanisms of action, modes of delivery, and efficacy against coronaviruses. Am J Infect Control. (2020) 48:1062–7. 10.1016/j.ajic.2020.06.182 32565272 PMC7301780

[54] Chen AP Chu IY Yeh ML Chen YY Lee CL Lin HH. Differentiating impacts of non-pharmaceutical interventions on non-coronavirus disease-2019 respiratory viral infections: Hospital-based retrospective observational study in Taiwan. Influenza Other Respir Viruses. (2021) 15:478–87. 10.1111/irv.12858 33825310 PMC8189242

[55] Kume Y Hashimoto K Chishiki M Norito S Suwa R Ono T. Changes in virus detection in hospitalized children before and after the severe acute respiratory syndrome coronavirus 2 pandemic. Influenza Other Respir Viruses. (2022) 16:837–41. 10.1111/irv.12995 35488324 PMC9343337

[56] Hills T Kearns N Kearns C Beasley R. Influenza control during the COVID-19 pandemic. Lancet. (2020) 396:1633–4. 10.1016/S0140-6736(20)32166-8 33228919 PMC7581384

[57] Chu IY-H Alam P Larson HJ Lin L. Social consequences of mass quarantine during epidemics: a systematic review with implications for the COVID-19 response. J Travel Med. (2020) 27:1–14. 10.1093/jtm/taaa192 33051660 PMC7649384

[58] Davis B Rothrock AN Swetland S Andris H Davis P Rothrock SG. Viral and atypical respiratory co-infections in COVID-19: a systematic review and meta-analysis. J Am College Emergency Physicians Open. (2020) 1:533–48. 10.1002/emp2.12128 32838380 PMC7323310

[59] Nickbakhsh S Ho A Marques DFP McMenamin J Gunson RN Murcia PR. Epidemiology of seasonal coronaviruses: establishing the context for the emergence of Coronavirus Disease 2019. J Infect Dis. (2020) 222:17–25. 10.1093/infdis/jiaa185 32296837 PMC7184404

[60] Paret M Lalani K Hedari C Jaffer A Narayanan N Noor A. SARS-CoV-2 Among Infants < 90 Days of Age Admitted for Serious Bacterial Infection Evaluation. Pediatrics. (2021) 148:4465. 10.1542/peds.2020-044685 34193619

[61] Shen DP. Impact of COVID-19 on viral respiratory infection epidemiology in young children: a single-center analysis [master's thesis on the Internet]. Leuven: KU Leuven (2022). Available online at: https://repository-teneo-libis-be.kuleuven.e-bronnen.be/delivery/DeliveryManagerServlet?dps_pid=IE16696915& 10.3389/fpubh.2022.931242 PMC9530989 36203684

