# Supplementary figures and images for: Impact of COVID-19 on viral respiratory infection epidemiology in young children: A single-center analysis
Source: Front Public Health. 2022 Sep 20;10:931242. doi: 10.3389/fpubh.2022.931242 (PMC9530989; doi:10.3389/fpubh.2022.931242)

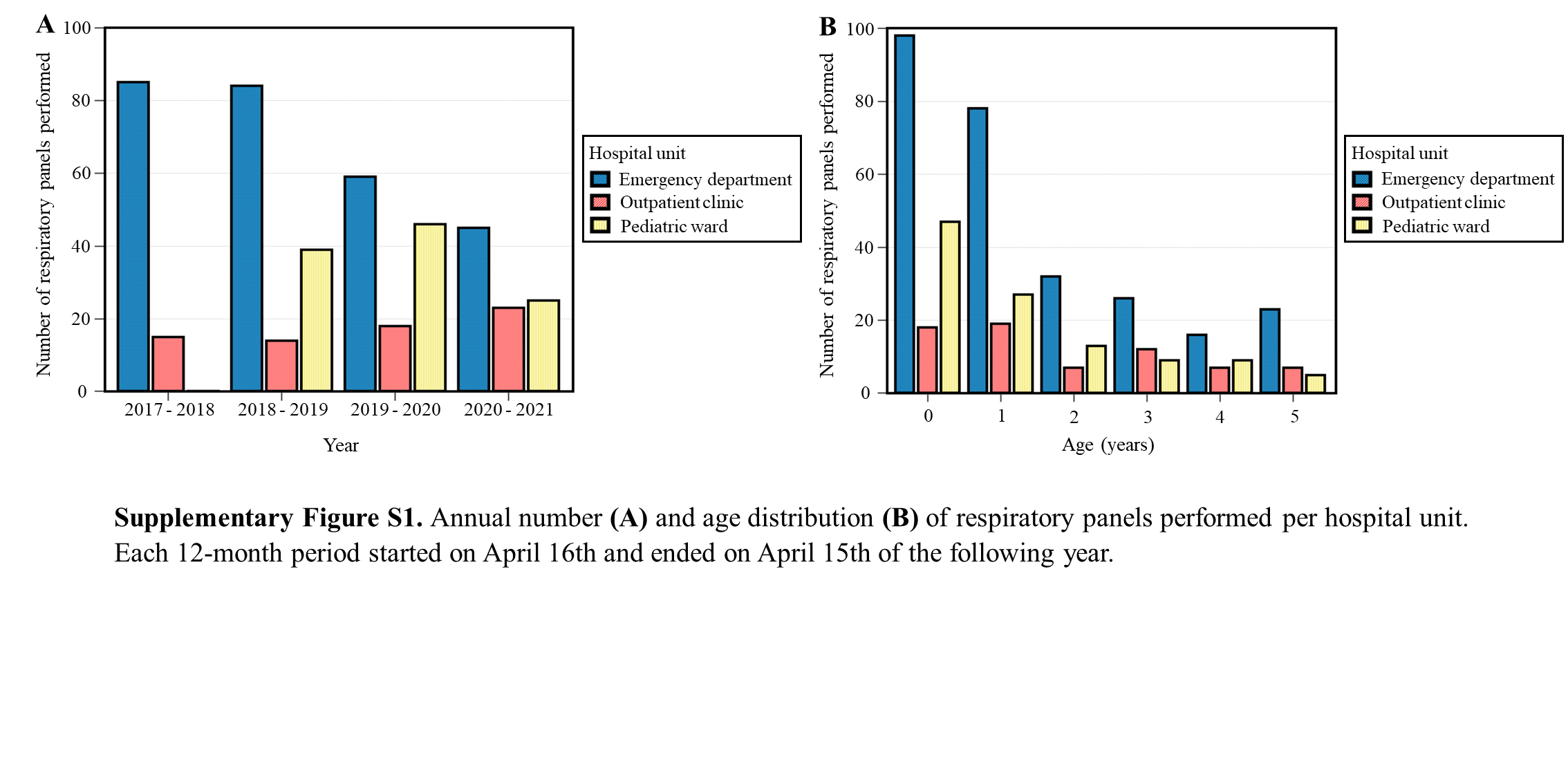

Supplement: Supplementary file 1 [file Image_1.TIF]

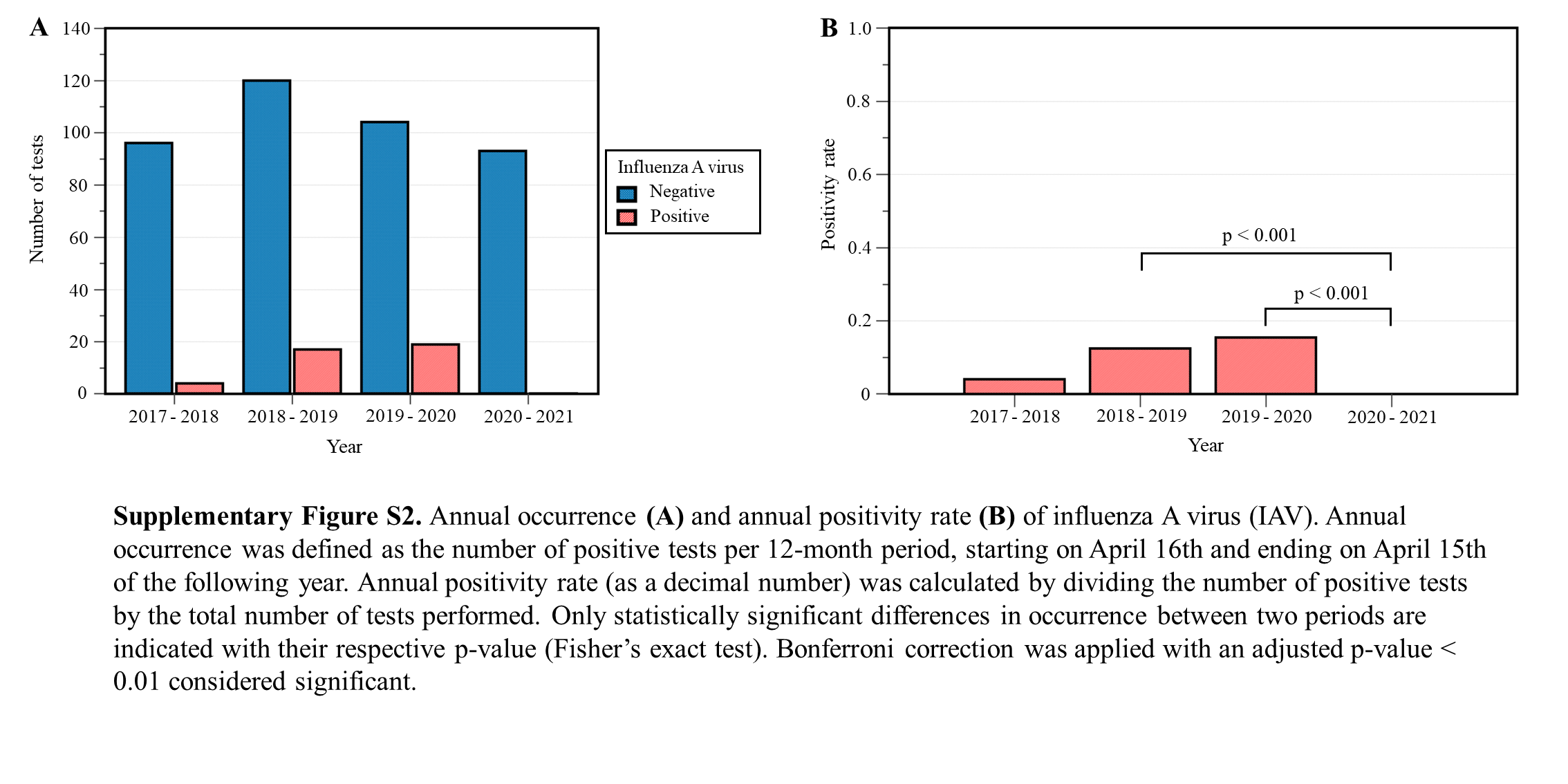

Supplement: Supplementary file 2 [file Image_2.TIF]

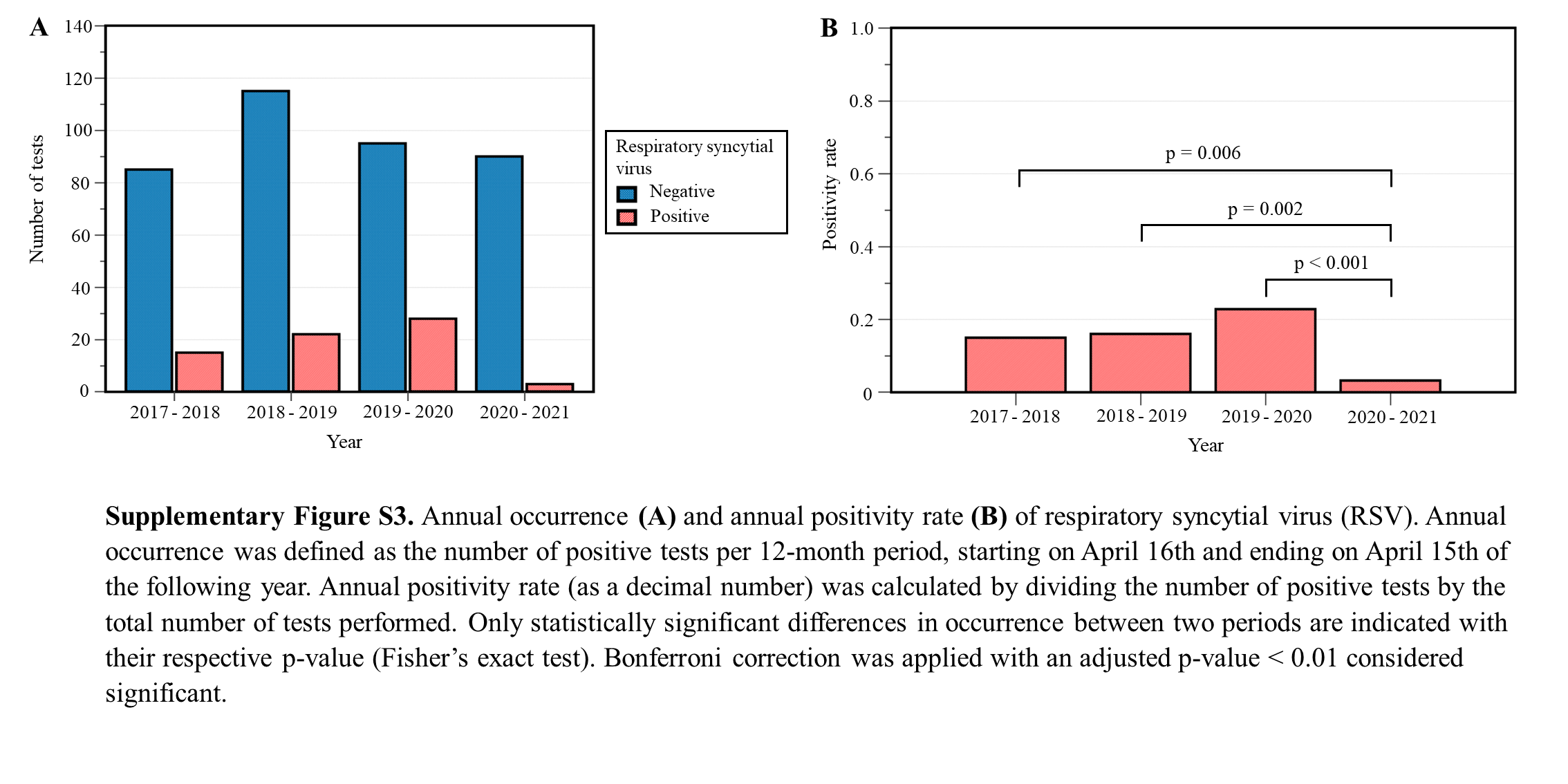

Supplement: Supplementary file 3 [file Image_3.TIF]

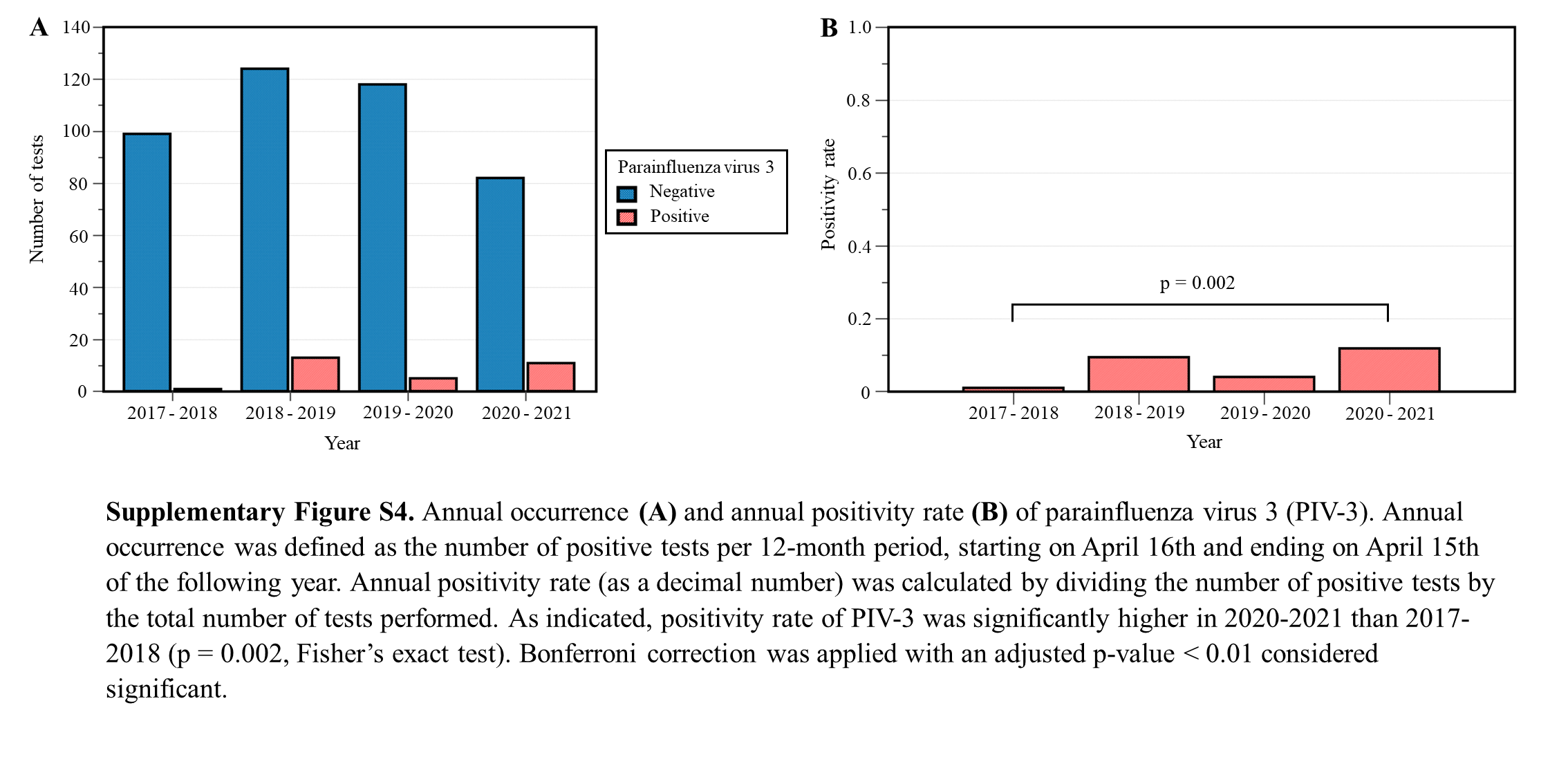

Supplement: Supplementary file 4 [file Image_4.TIF]

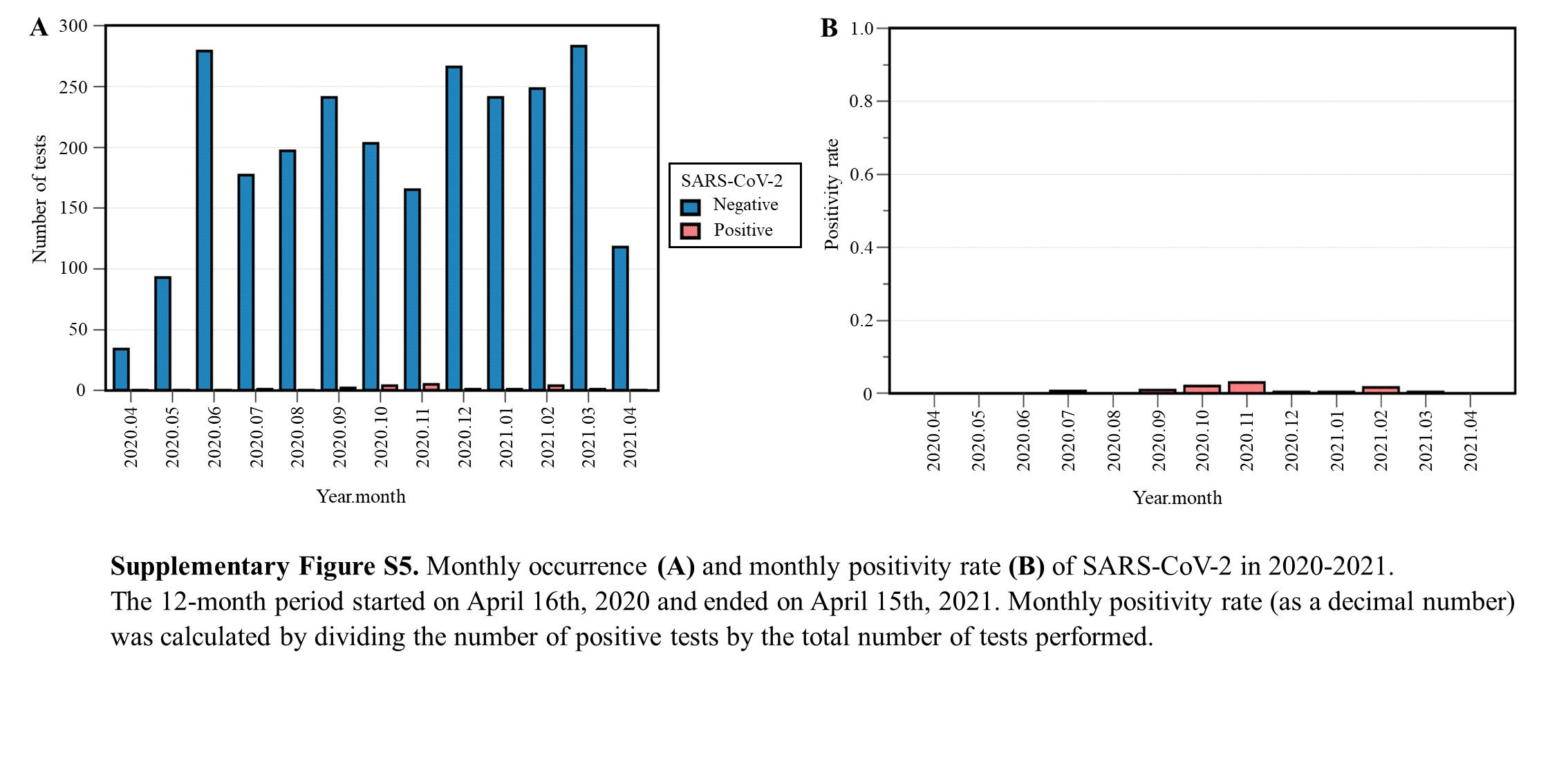

Supplement: Supplementary file 5 [file Image_5.TIF]

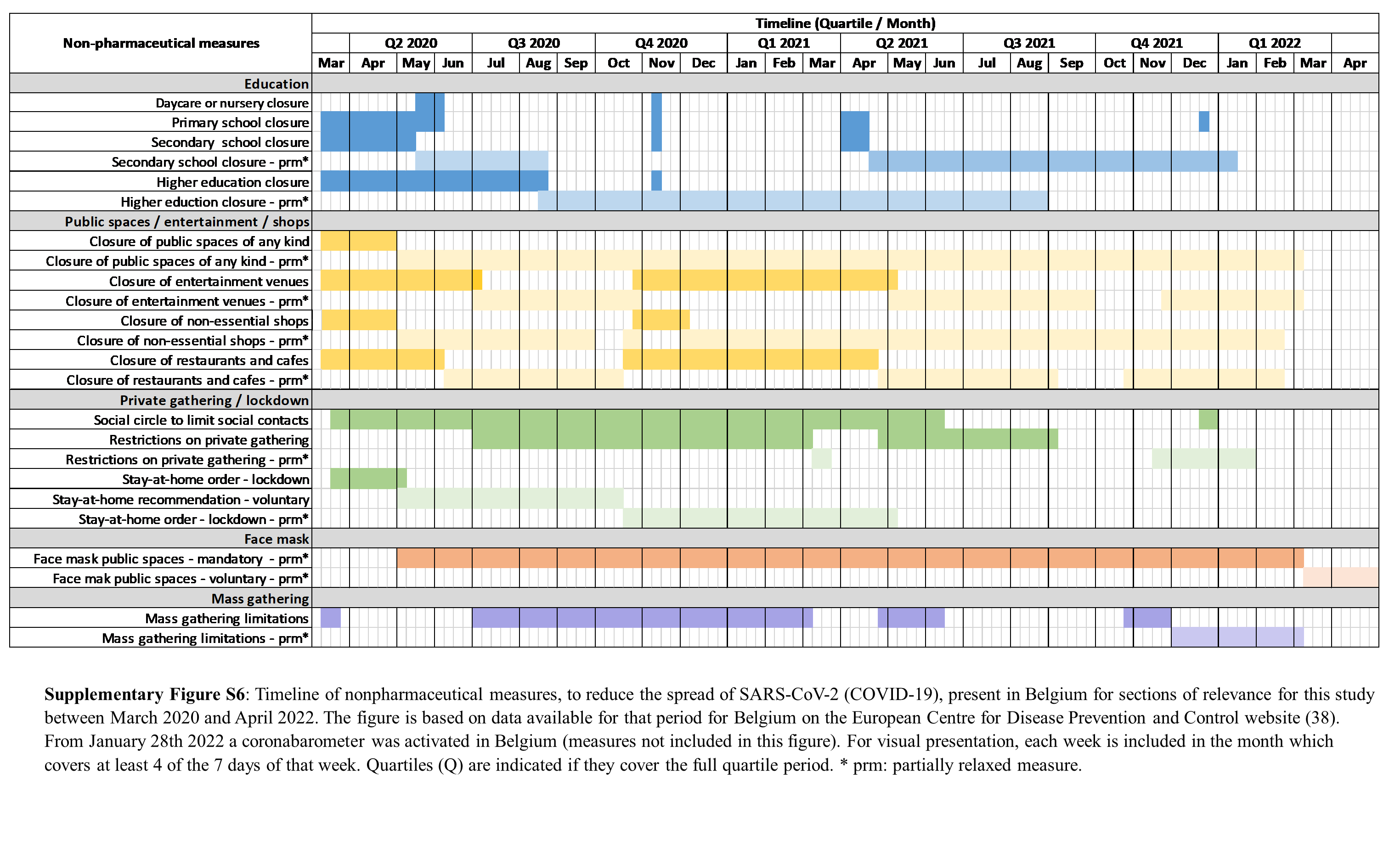

Supplement: Supplementary file 6 [file Image_6.TIF]
