# Supplementary material for: Impact of COVID-19 on viral respiratory infection epidemiology in young children: A single-center analysis
Source: Front Public Health. 2022 Sep 20;10:931242. doi: 10.3389/fpubh.2022.931242 (PMC9530989; doi:10.3389/fpubh.2022.931242)
Supplement: Supplementary file 7 [file Table_1.pdf]

	Age category											
	0 years old		1 years old		2 years old		3 years old		4 years old		5 years old	
**Respiratory pathogen**	**Number of positive tests**	**Percentage of total positive tests***	**Number of positive tests**	**Percentage of total positive tests***	**Number of positive tests**	**Percentage of total positive tests***	**Number of positive tests**	**Percentage of total positive tests***	**Number of positive tests**	**Percentage of total positive tests***	**Number of positive tests**	**Percentage of total positive tests***
Influenza A virus	6	1.6%	7	2.3%	11	8.2%	7	7.4%	4	6.8%	5	7.6%
Influenza B virus	0	0.0%	1	0.3%	1	0.7%	2	2.1%	2	3.4%	0	0.0%
Respiratory syncytial virus	32	8.6%	22	7.3%	5	3.7%	4	4.2%	2	3.4%	3	4.5%
Parainfluenza virus 1	2	0.5%	3	1.0%	0	0.0%	1	1.1%	0	0.0%	1	1.5%
Parainfluenza virus 2	2	0.5%	1	0.3%	0	0.0%	0	0.0%	0	0.0%	0	0.0%
Parainfluenza virus 3	8	2.1%	13	4.3%	5	3.7%	1	1.1%	1	1.7%	2	3.0%
Parainfluenza virus 4	2	0.5%	1	0.3%	0	0.0%	2	2.1%	1	1.7%	0	0.0%
Adenovirus	33	8.8%	36	12.0%	9	6.7%	2	2.1%	4	6.8%	2	3.0%
Bocavirus	18	4.8%	12	4.0%	5	3.7%	2	2.1%	1	1.7%	0	0.0%
Human metapneumovirus	8	2.1%	3	1.0%	5	3.7%	2	2.1%	2	3.4%	1	1.5%
Human rhinovirus/enterovirus	86	23.0%	53	17.7%	27	20.1%	18	18.9%	10	16.9%	10	15.2%
Coronavirus 229E	1	0.3%	0	0.0%	0	0.0%	0	0.0%	0	0.0%	1	1.5%
Coronavirus HKU-1	7	1.9%	1	0.3%	0	0.0%	0	0.0%	1	1.7%	0	0.0%
Coronavirus NL63	0	0.0%	4	1.3%	2	1.5%	0	0.0%	0	0.0%	1	1.5%
Coronavirus OC43	4	1.1%	5	1.7%	0	0.0%	1	1.1%	0	0.0%	1	1.5%
SARS-CoV-2	6	1.6%	3	1.0%	2	1.5%	5	5.3%	1	1.7%	3	4.5%
Chlamydophila pneumoniae	0	0.0%	2	0.7%	0	0.0%	1	1.1%	2	3.4%	0	0.0%
Chlamydophila psittaci	0	0.0%	0	0.0%	0	0.0%	0	0.0%	0	0.0%	0	0.0%
Coxiella burnetii	0	0.0%	0	0.0%	0	0.0%	0	0.0%	0	0.0%	0	0.0%
Cytomegalovirus	36	9.6%	44	14.7%	18	13.4%	14	14.7%	4	6.8%	1	1.5%
Enterovirus D68	0	0.0%	1	0.3%	0	0.0%	0	0.0%	1	1.7%	0	0.0%
Herpes simplex virus 1	3	0.8%	3	1.0%	8	6.0%	2	2.1%	2	3.4%	3	4.5%
Herpes simplex virus 2	0	0.0%	0	0.0%	0	0.0%	0	0.0%	0	0.0%	0	0.0%
Legionella pneumophila	0	0.0%	0	0.0%	0	0.0%	0	0.0%	0	0.0%	0	0.0%
MERS-coronavirus	0	0.0%	0	0.0%	0	0.0%	0	0.0%	0	0.0%	0	0.0%
Mycoplasma pneumoniae	3	0.8%	1	0.3%	0	0.0%	0	0.0%	0	0.0%	4	6.1%
Parechovirus	7	1.9%	10	3.3%	2	1.5%	0	0.0%	0	0.0%	1	1.5%
Pneumocystis jirovecii	24	6.4%	5	1.7%	2	1.5%	0	0.0%	0	0.0%	0	0.0%
SARS-associated coronavirus	0	0.0%	0	0.0%	0	0.0%	0	0.0%	0	0.0%	0	0.0%
Streptococcus pneumoniae	86	23.0%	69	23.0%	32	23.9%	31	32.6%	21	35.6%	27	40.9%
**Total positive tests***	**374**		**300**		**134**		**95**		**59**		**66**	

Supplementary Table S1. Distribution of respirtory pathogens during the study period for the different ages. Listed in the table are the number of positive tests and percentage of total positive tests for each respiratory pathogen over the entire study period, per age category. “Total of positive tests” (*) per age category concerns the sum of all positive tests, comprising all respiratory pathogens, in this respective age category.
